# Choroidal vascular biomarkers for macular neovascularization in chronic central serous chorioretinopathy: insights from widefield indocyanine green angiography

**DOI:** 10.1186/s40662-026-00494-0

**Published:** 2026-06-09

**Authors:** Xinlei Hao, Jiaxin Pu, Yuhong Gan, Yongyue Su, Xuenan Zhuang, Guiqin He, Yining Zhang, Xuelin Chen, Yi He, Guanghan Zhang, Miaoling Li, Feng Wen

**Affiliations:** State Key Laboratory of Ophthalmology, Guangdong Provincial Key Laboratory of Ophthalmology and Visual Science, Zhongshan Ophthalmic Center, Sun Yat-sen University, 54 South Xianlie Road, Guangzhou, 510060 China

**Keywords:** Chronic central serous chorioretinopathy, Macular neovascularization, Choroidal characteristics, Intervortex vein anastomosis, Widefield indocyanine green angiography

## Abstract

**Background:**

To investigate the correlation between choroidal vasculature characteristics and the formation of macular neovascularization (MNV) in patients with chronic central serous chorioretinopathy (CSC), based on widefield indocyanine green angiography (WF-ICGA).

**Methods:**

This was a retrospective, observational study. Patients with chronic CSC with complete medical records and multimodal imaging conducted between September 2022 and June 2025, were included. Eyes with chronic CSC were divided into the MNV and non-MNV groups based on the presence or absence of MNV. WF-ICGA was performed to obtain choroidal characteristics, including vortex vein (VV), dominant VV (DVV), posterior VV, intervortex venous anastomosis (IVA), and age-related scattered hypofluorescent spots on late-phase indocyanine green angiography (ASHS-LIA). Choroidal characteristics were quantitatively and qualitatively assessed and compared between eyes with and without MNVs.

**Results:**

A total of 293 eyes from 212 patients (164 males, 77.36%) were analyzed. Of these, 96 presented with secondary MNV (32.76%), whereas 197 did not. Compared with that of the non-MNV group, the MNV group exhibited a more advanced age and worse visual acuity (all *P* < 0.001). Regarding choroidal characteristics, MNV cases more frequently exhibited an asymmetric distribution pattern (*P* = 0.032), solitary DVV (*P* = 0.041), superotemporal DVV location (*P* = 0.005), macular IVA (*P* = 0.027), and ASHS-LIA (*P* < 0.001). In the generalized linear-mixed model, which was adjusted for age, sex, and symptom duration, the occurrence of MNV correlated with the presence of superotemporal DVV, macular IVA, and ASHS-LIA (*P* < 0.001, *P* = 0.003, and *P* = 0.011, respectively).

**Conclusion:**

WF-ICGA-derived choroidal vascular characteristics were identified as potential biomarkers for the development of MNV in eyes with chronic CSC. The presence of superotemporal DVV, macular IVA, and ASHS-LIA may serve as independent associated factors for predicting the development of MNV.

## Background

Central serous chorioretinopathy (CSC) represents one of the most prevalent vision-impairing retinopathies, following age-related macular degeneration (AMD), diabetic retinopathy, and retinal vein occlusion, which mainly manifests as local detachment of neurosensory retina in the macula [1–3]. As disease progresses, CSC exhibits significant diversity in its clinical manifestations; the acute phase of CSC is often self-limiting, whereby subretinal fluid (SRF) reabsorbs within 3 to 6 months, causing little compromise to retinal integrity [2, 4]. However, chronic CSC involves progressive, diffuse changes within the retinal pigment epithelium (RPE) and choroid, carrying a significant risk of complications such as macular neovascularization (MNV) [4]. The identification of MNV dictates a shift in clinical strategy, necessitating prompt anti-vascular endothelial growth factor (VEGF) therapy instead of conservative management or laser photocoagulation [5]. MNV secondary to CSC is commonly classified as type 1 MNV, characterized by hyperreflective, flat, and irregular RPE elevation on optical coherence tomography (OCT) [5, 6]. The occurrence of MNV typically compromises visual prognosis [5, 7, 8]. Therefore, understanding the pathogenesis of CSC-associated MNV remains a critical research priority.

Previous studies have extensively analyzed diverse risk factors for MNV secondary to CSC, including clinical features and imaging biomarkers [4, 9]. These factors encompassed sex, age, duration, subtype, best-corrected visual acuity (BCVA), and inner choroidal attenuation, et al. [4, 8, 9]. Recently, the venous overload hypothesis has emerged as a major focus in the field of CSC pathogenesis research, involving delayed choriocapillaris filling, choroidal vascular hyperpermeability (CVH), asymmetric drainage pathways, and intervortex venous anastomoses [10–13]. Recent advances in multimodal imaging have substantially refined our understanding of the pachychoroid disease spectrum, highlighting the pivotal roles of choroidal structural remodeling [14]. The evolution of widefield imaging technology has revolutionized choroidal evaluation, simultaneously providing mechanistic insights into CSC pathogenesis [15]. Cennamo et al. [16] suspected that choroidal hypoperfusion may correlate with the development of MNV among patients with CSC, which paralleled with other neovascular chorioretinal disorders [17]. Bacci et al. [18] have investigated that imbalanced choroidal venous pattern and congestion of certain vortex vein (VV) may cause choroidal venous insufficiency among pachychoroid diseases using ultra-widefield indocyanine green angiography (UWF-ICGA). Based on widefield indocyanine green angiography (WF-ICGA), our team’s earlier investigation demonstrated that CSC cases with a single dominant VV (DVV) distribution exhibited lower overall choriocapillaris flow density (CCFD) relative to that of those with symmetrical configuration or with two DVVs pattern [13].

Given this potential link between choroidal features and MNV development, we used WF-ICGA to assess the correlation between choroidal vasculature and the formation of MNV in patients with chronic CSC, with an aim to find choroidal imaging biomarkers to guide risk stratification and personalized follow-up surveillance.

## Methods

### Study design and participants

This retrospective, observational study protocol was approved by the ethics committee of Zhongshan Ophthalmic Center (2023KYPJ114) and adhered to the tenets of the Declaration of Helsinki. Informed consent was obtained from all participants included in the study.

Consecutive patients presenting to Zhongshan Ophthalmic Center between September 2022 and June 2025 with a clinical diagnosis of chronic CSC, who underwent thorough ophthalmic assessment, were retrospectively analyzed. The diagnosis of CSC was based on major and minor criteria in accordance with multimodal imaging-based classification [19]. Chronic CSC was characterized by persistent visual symptoms along with documented SRF and RPE alterations for longer than 6 months [4, 20]. Exclusion criteria included: (1) lack of clinical records or multimodal imaging results, especially the absence of early- and late-phase angiographic images, making it impossible to obtain choroidal characteristics; (2) any concomitant chorioretinal disorders affecting clinical and imaging judgment, including polypoidal choroidal vasculopathy (PCV), intraocular inflammatory diseases, and choroidal tumors; and (3) media opacity or poor fixation compromising the quality of images.

### Imaging protocol

Inclusion was limited to patients who underwent a comprehensive standardized ophthalmic evaluation, including BCVA (Snellen chart), intraocular pressure (IOP) (Topcon CT-1, Topcon, Japan), slit-lamp examination, and indirect ophthalmoscopy. Snellen BCVA was converted into logarithmic minimal angle of resolution (logMAR) for statistical analysis. Multimodal imaging protocol included color fundus photography (Clarus 500, Zeiss, Germany or aTRC-50X, Topcon, Japan), OCT (Spectralis OCT, Heidelberg Engineering, Germany), wide-field optical coherence tomography angiography (WF-OCTA; TowardPi BM400K BMizar, TowardPi Medical Technology, China), fundus fluorescein angiography (FFA), and WF-ICGA (Spectralis HRA, Heidelberg Engineering, Germany).

WF-ICGA enabled single-frame visualization of the choroid up to 102-degree field of view. In this study, early- and mid-phase images were acquired using wide-field imaging (102°), whereas late-phase angiograms were obtained using a conventional 55-degree range. Early-phase imaging was performed continuously within the first 5 min following dye injection, whereas mid-phase images were obtained between 8 and 15 min post-injection. For each patient, the angiograms were extended beyond 30 min to record late-phase results. A standardized nine-point fixation protocol covering the fovea and eight peripheral positions (superior, superotemporal, temporal, inferotemporal, inferior, inferonasal, nasal, and superonasal) was used to capture the entire choroidal vasculature.

### Grouping

**Fig. 1:**
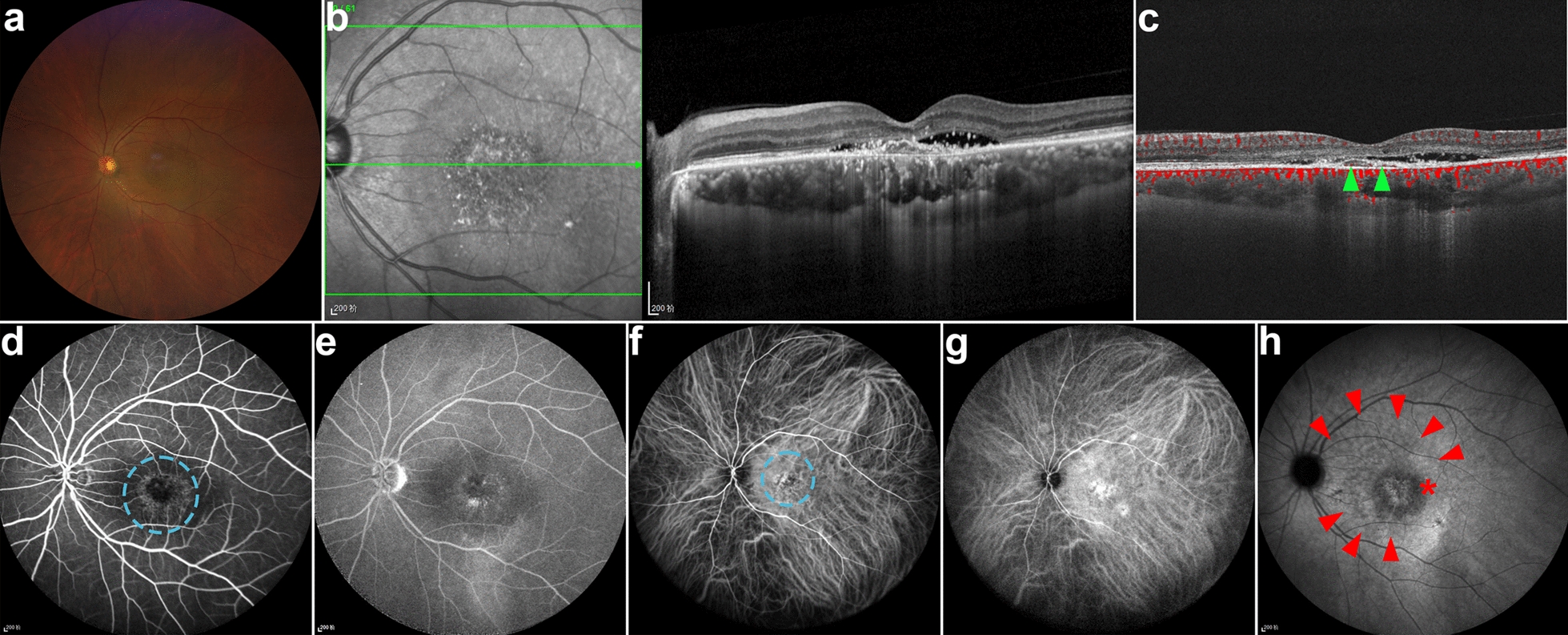
Multimodal imaging of a representative chronic central serous chorioretinopathy (CSC) eye with secondary type 1 macular neovascularization (MNV). **a** Color fundus photography showed irregular pigment derangement in the macular region with an inferiorly extending shallow neurosensory detachment. **b** Near-infrared images and corresponding optical coherence tomography (OCT) revealed heterogeneous, flat, irregular pigment epithelium detachment (FIPED) and subretinal fluid (SRF). **c** OCT angiography B-scan exhibited SRF and FIPED with blood flow signal (green arrowheads). **d**, **e** Early-phase fundus fluorescein angiography demonstrated scattered hyperfluorescence in the macular region (**d**, blue dotted line), while late-phase imaging showed progressive staining and leakage (**e**). **f**–**h** Widefield indocyanine green angiography showed ill-defined vascular network within the macula in the early-phase (**f**, blue dotted line), which evolved into patchy hyperfluorescence with indistinct margin during the mid-phase (**g**) and culminated in staining lesion in the late-phase (**h**, red asterisk). Additionally, age-related scattered hypofluorescent spots on late-phase indocyanine green angiography was also observed which was marked as red arrowheads

Eyes with chronic CSC were divided into MNV and non-MNV groups based on the presence or absence of type 1 MNVs. Based on multimodal imaging examinations, MNV was considered present when the following diagnostic criteria were fulfilled (Fig. 1): (1) heterogeneous, flat, irregular pigment epithelium detachment (FIPED) on OCT; (2) neovascular complex detected on en face WF-OCTA images from the outer retinal to choriocapillaris slab and blood flow signal discerned from corresponding B-scan images; and (3) ill-defined hyperfluorescent lesions with late staining on FA and WF-ICGA. It should be noted that the diagnosis of MNV requires fulfillment of at least the first two criteria, whereas the third criterion serves as supportive evidence.

### Definition of choroidal characteristics

Early-phase WF-ICGA imaging was performed to qualitatively or quantitatively assess various choroidal features in the two groups, including VV, DVV, posterior VV (PVV), and intervortex venous anastomosis (IVA). Late-phase imaging was used to identify and grade age-related scattered hypofluorescent spots on late-phase indocyanine green angiography (ASHS-LIA) among enrolled eyes.

**Fig. 2:**
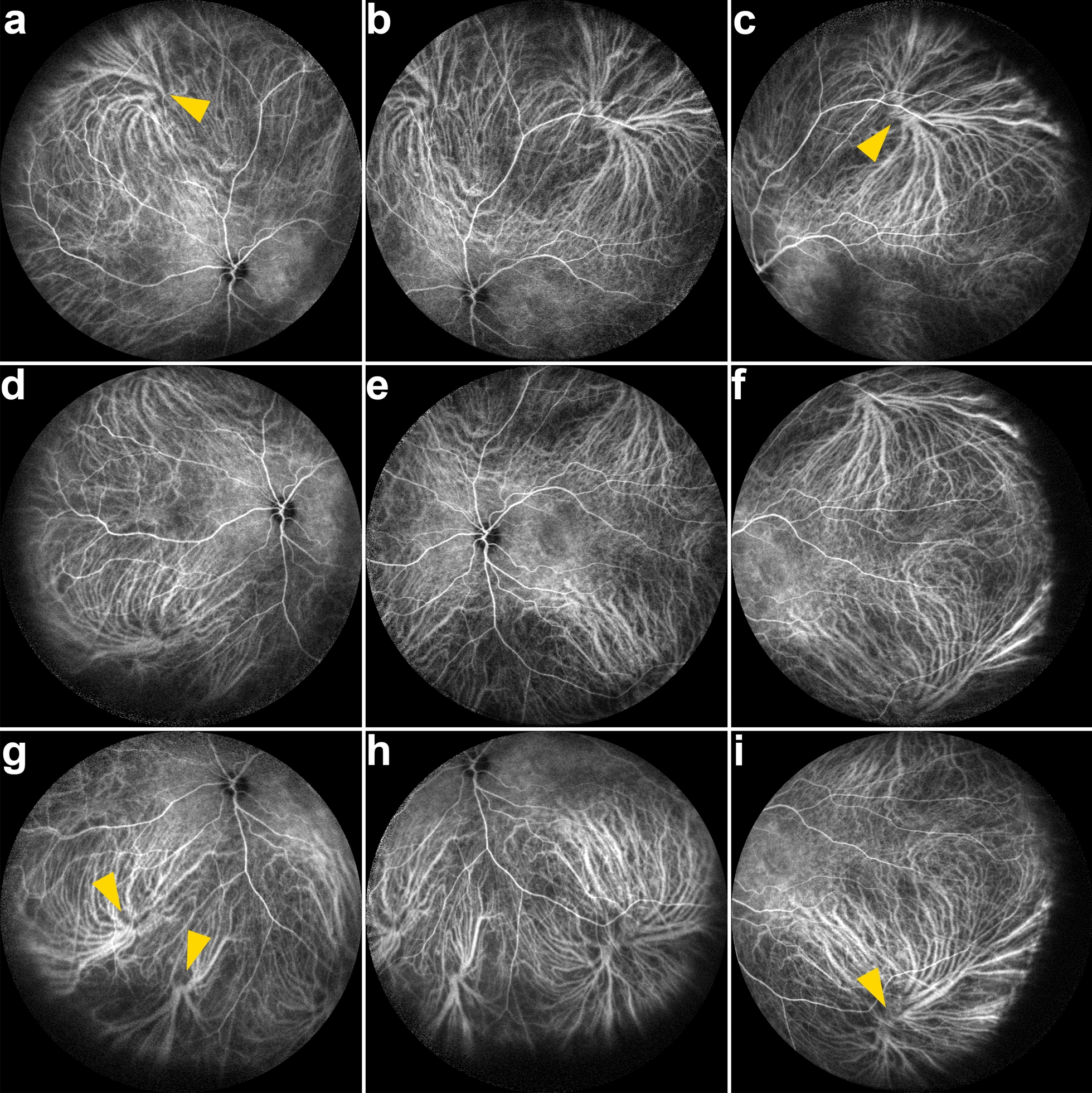
Diagram illustrating the methodology for quantifying vortex vein ampulla using early-phase widefield indocyanine green angiography. Images were acquired from superonasal (**a**), superior (**b**), superotemporal (**c**), nasal (**d**), fovea (**e**), temporal (**f**), inferonasal (**g**), inferior (**h**), and inferotemporal (**i**) orientations. Each vortex vein ampulla was marked as yellow arrowheads

Hayreh confirmed the existence of physiological watershed zones between VVs in rhesus monkeys, with FFA imaging revealing cruciform hypofluorescence in the choriocapillaris [21]. The horizontal watershed zone crossed the macular region and optic disc, and the vertical watershed zone passed through the temporal side of the papilla, thereby partitioning the choroidal venous drainage into four independent quadrants (superotemporal, superonasal, inferotemporal, and inferonasal), with each VV residing in its own distinct quadrant [21]. The number of VV ampulla was recorded for each quadrant, and the total count per affected eye was determined (Fig. 2).

**Fig. 3:**
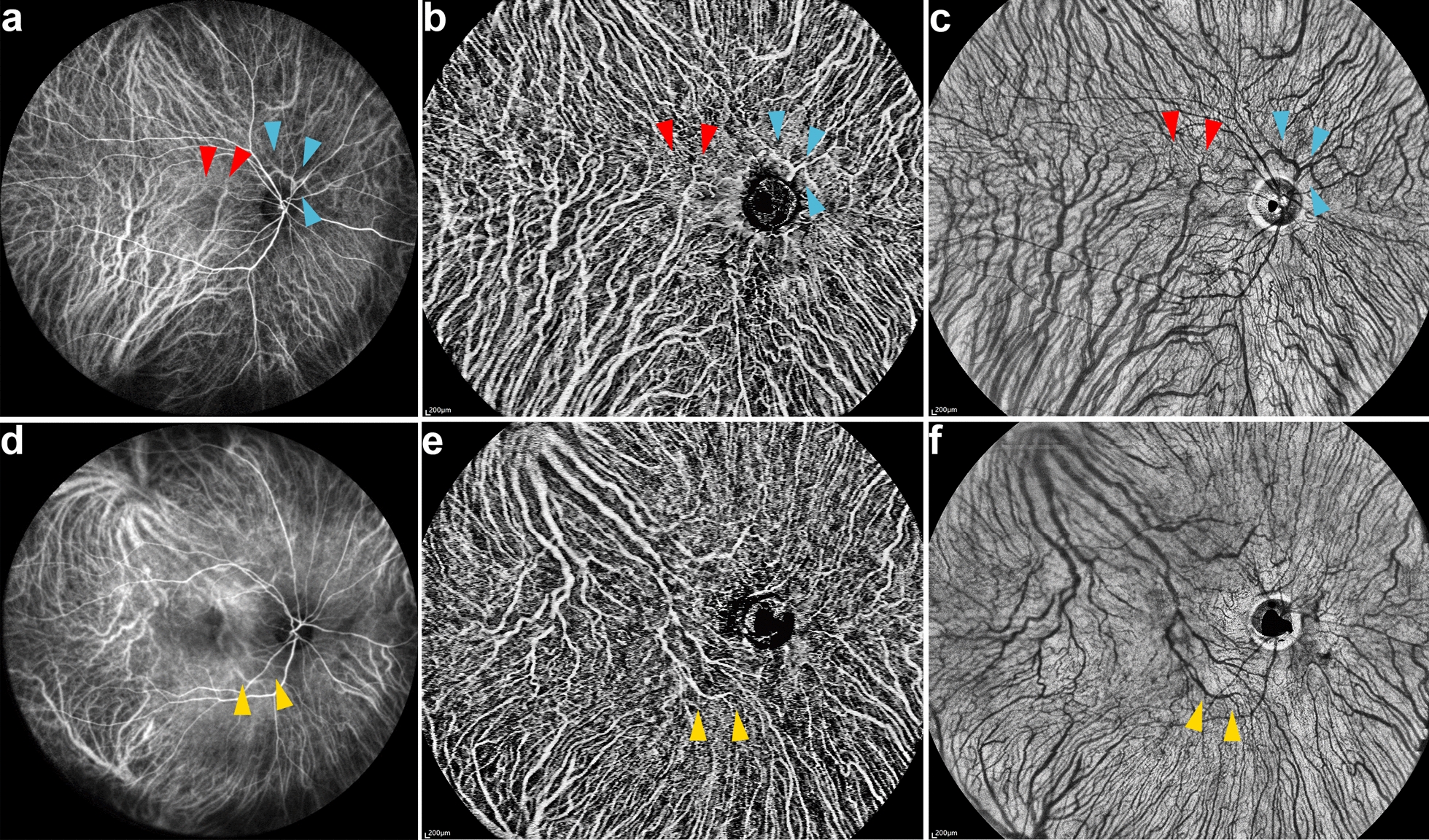
Representative case presentation of dominant vortex vein (DVV) and posterior vortex vein (PVV) anatomy. **a**–**c** Widefield indocyanine green angiography (WF-ICGA), widefield optical coherence tomography angiography (WF-OCTA), and widefield en face optical coherence tomography (OCT) showed the presence of inferotemporal DVV (red arrowheads) and PVV (blue arrowheads). **d**–**f** WF-ICGA, WF-OCTA and widefield en face OCT showed the presence of superotemporal DVV (yellow arrowheads)

Using the fovea and optic disc center as anatomical landmarks, a DVV was identified when the termination of the lateral temporal VV traversed the macular fovea or the termination of the lateral nasal VV crossed the optic disc center (Fig. 3) [13]. The remaining VVs were considered as non-DVVs. The presence of a DVV drainage route indicated an asymmetric distribution pattern, otherwise, it was symmetric. The number and location of the DVVs were individually recorded.

PVV were defined as larger choroidal vessels that collected blood from the choriocapillaris and exited the eyeball in the posterior pole (Fig. 3) [22].

**Fig. 4:**
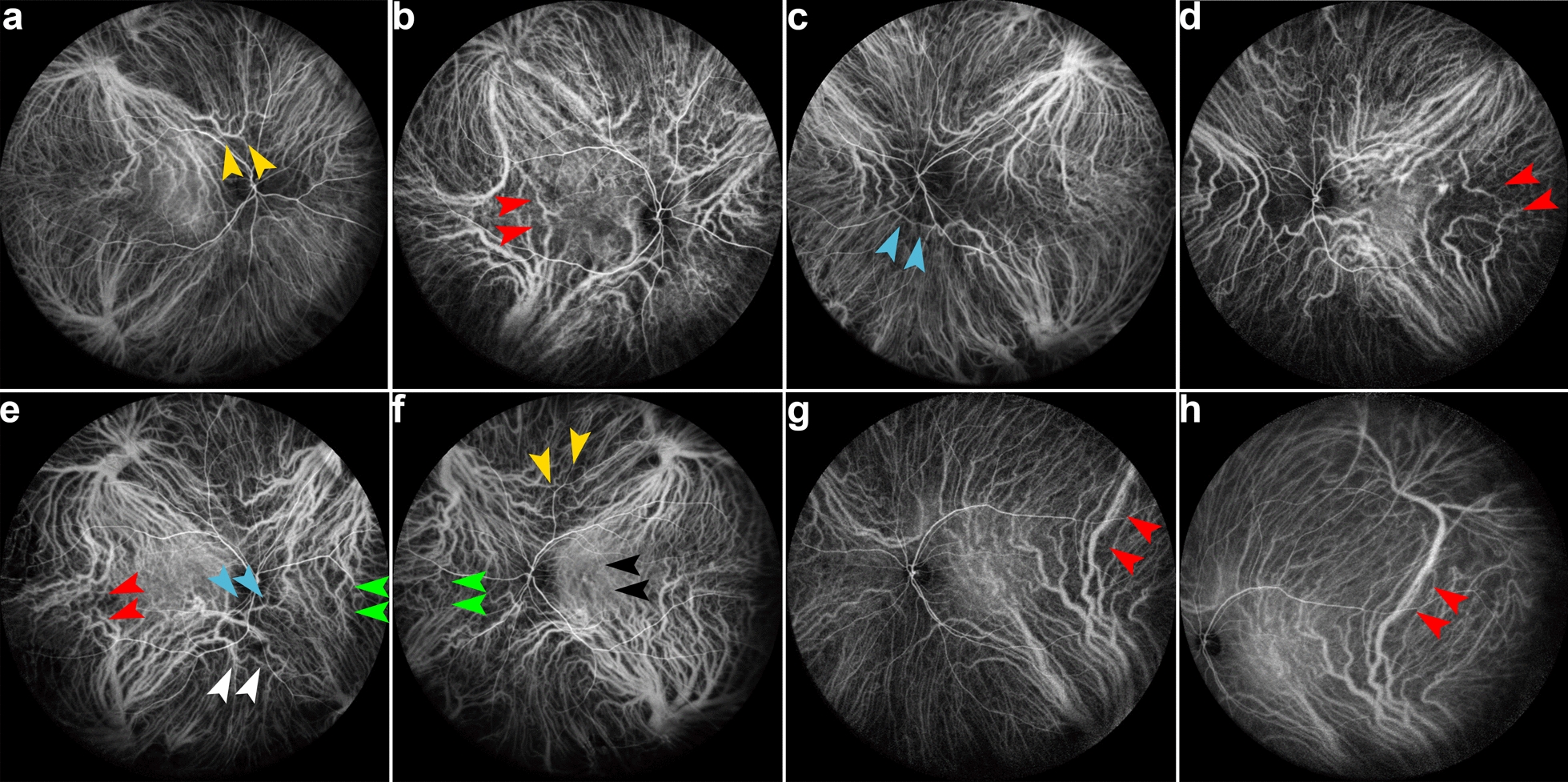
Representative case presentation of intervortex venous anastomosis (IVA) anatomy utilizing early-phase widefield indocyanine green angiography imaging. **a** Superior IVA was present connecting superotemporal and superonasal vortex vein drainage systems (yellow arrowheads). **b** Temporal IVA was present connecting superotemporal and inferotemporal vortex vein drainage systems (red arrowheads). **c** Peripapillary IVA was present connecting superonasal and inferotemporal vortex vein drainage systems (blue arrowheads). **d** Temporal IVA was present connecting superotemporal and inferotemporal vortex vein drainage systems (red arrowheads). **e** A single eye exhibited numerous anastomotic connections between vortex vein systems: temporal IVA connecting superotemporal and inferotemporal drainage systems (red arrowheads), inferior IVA connecting inferotemporal and inferonasal drainage systems (white arrowheads), nasal IVA connecting superonasal and inferonasal drainage systems (green arrowheads), and peripapillary IVA connecting inferotemporal and superonasal drainage systems (blue arrowheads). **f** A single eye exhibited numerous anastomotic connections between vortex vein systems: superior IVA connecting superotemporal and superonasal drainage systems (yellow arrowheads), nasal IVA connecting superonasal and inferonasal drainage systems (green arrowheads), and macular IVA connecting superotemporal and inferotemporal drainage systems (black arrowheads). **g**, **h** The complete course of temporal anastomotic branches could not be fully delineated from macula-centered widefield images alone (**g**); thus, widefield acquisitions with superotemporal fixation (**h**) were required to confirm the presence of a temporal IVA (red arrowheads), which connected superotemporal and inferotemporal drainage systems

Anastomosis between the VV systems was confirmed when two or more connecting vessels were present linking adjacent quadrants [23]. Classification of IVAs was based on their anatomical location, yielding three distinct categories: macular, peripapillary, and peripheral IVA (Fig. 4). A macular IVA was identified when the anastomotic vessel traversed the macular region (within the range of the temporal vascular arch). Peripapillary IVA was defined as its presence within one disc diameter of the optic disc. Other configurations were classified as peripheral IVA, which were further subdivided into temporal, superior, nasal, and inferior IVA subtypes. The temporal IVA, temporal to the macular region, crosses the horizontal watershed zone to connect the superotemporal and inferotemporal quadrants; the nasal IVA, situated nasally to the optic disc, also traverses the horizontal zone, bridging the superonasal and inferonasal quadrants; the superior IVA, located above the optic disc, penetrates the vertical watershed zone, thereby uniting the superotemporal and superonasal quadrants; and the inferior IVA, below the disc, crosses the vertical zone to link the inferotemporal and inferonasal quadrants.

**Fig. 5:**
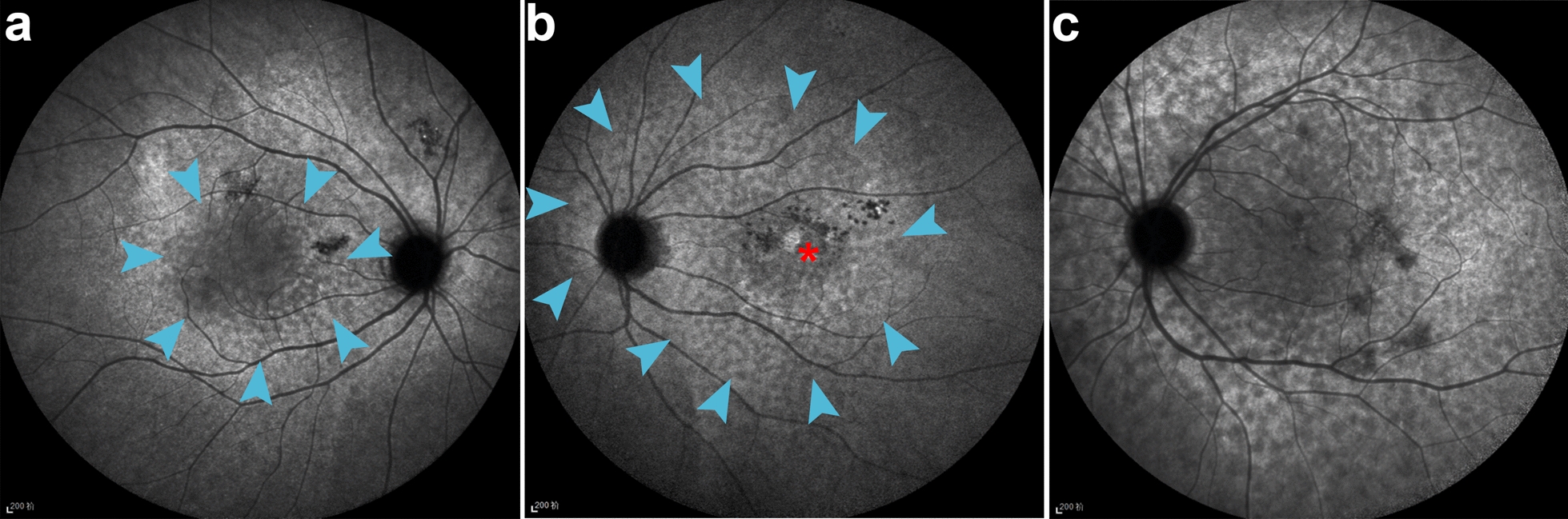
Representative case presentation of age-related scattered hypofluorescent spots on late-phase indocyanine green angiography (ASHS-LIA). **a** In an eye with chronic central serous chorioretinopathy (CSC), ASHS-LIA was classified as Grade 1, confined to the macula (blue arrowheads). **b** In an eye with chronic CSC-associated macular neovascularization, ASHS-LIA was classified as Grade 2, extending to both the macula and peripapillary region (blue arrowheads). Late staining of the neovascular lesion can be observed (red asterisk). **c** In an eye with chronic CSC, ASHS-LIA was classified as Grade 3, diffusely scattered throughout the posterior pole

ASHS-LIA manifested as discrete hypofluorescent spots on late-phase ICGA images (Fig. 5), which were graded based on the extent of involvement as follows: Grade 1 (macular region), Grade 2 (macular region and around the optic disc), and Grade 3 (throughout the posterior pole) [24].

Two independent and experienced ophthalmologists (X.H. and J.P.) diagnosed the enrolled eyes and evaluated the images. When consensus could not be reached between the two investigators, a third retinal specialist (F. W.) made the final decision.

### Statistical analysis

Statistical analyses were performed using SPSS software (version 26.0, SPSS Inc., USA), with a significance level set at *P* < 0.05. Continuous variables were presented as mean ± standard deviation (SD) or median and interquartile range (IQR). Categorical variables were summarized as frequencies and percentages. The Shapiro–Wilk test was performed to validate the results of the normality assumption. To explore the differences in demographic features and choroidal characteristics between the MNV and non-MNV groups, statistical analyses were conducted using independent sample t-test, Mann–Whitney U test, chi-square test and Fisher’s exact test, as appropriate, for the variable type and distribution mode. To account for the correlation between the eyes of the same patient, a generalized linear-mixed model was applied to determine the relationship between choroidal vasculature characteristics and MNV presence in chronic CSC cases. Interobserver agreement was conducted for the assessment of WF-ICGA features, and the kappa coefficient was used for the categorical evaluation of the location of the DVV and the presence of the PVV, IVA, and ASHS-LIA, while the intraclass correlation coefficient (ICC) was employed for the continuous variable of the VV ampulla number.

## Results

### General features of the enrolled patients

A total of 293 eyes of 212 patients with chronic CSC met the inclusion criteria. The demographic and clinical features of the patients are summarized in Table 1. The cohort included 164 male patients (77.36%), and their mean ± SD age was 49.24 ± 9.18 years. Of the included eyes, the mean duration of symptoms ± SD was 32.76 ± 30.85 months. Overall, the mean BCVA ± SD of the enrolled eyes was 0.44 ± 0.43 logMAR, with a mean spherical equivalent ± SD of 0.40 ± 1.03 D.

**Table 1:** Demographic of enrolled patients with chronic central serous chorioretinopathy

Demographic	Total	Non-MNV group	MNV group	*P* value^*^
No. of patients	212	–	–	–
Sex, M/F, n (%)	164/212 (77.36)/ 48/212 (22.64)	–	–	–
No. of Eyes	293	197	96	–
OD, n (%)	145/293 (49.49)	91/197 (46.19)	54/96 (56.25)	0.106^‡^
OS, n (%)	148/293 (50.51)	106/197 (53.81)	42/96 (43.75)	
Age (years) mean ± SD (range)	49.24 ± 9.18 (22–74)	48.26 ± 9.06 (22–74)	52.22 ± 8.82 (35–73)	** < 0.001**^†^
Duration of symptom (months) mean ± SD (range)	32.76 ± 30.85 (7–120)	31.35 ± 31.25 (7–120)	35.66 ± 29.97 (7–120)	0.263^†^
Spherical equivalent (diopters) mean ± SD (range)	0.40 ± 1.03 (− 2.75–2.875)	0.43 ± 1.00 (− 2.75–2.75)	0.35 ± 1.09 (− 2.625–2.875)	0.609^†^
BCVA (logMAR) mean ± SD (range)	0.44 ± 0.43 (− 0.1–2.0)	0.33 ± 0.37 (− 0.1–2.0)	0.63 ± 0.47 (0.0–2.0)	** < 0.001**^†^
IOP (mmHg) mean ± SD (range)	14.40 ± 2.20 (11.00–20.00)	14.42 ± 2.19 (11.00–20.00)	14.37 ± 2.24 (11.00–18.30)	0.868^†^
Previous treatments, n (%)				
Photodynamic therapy	6/293 (2.05)	3/197 (1.52)	3/96 (3.12)	0.715^§^
Laser therapy	65/293 (22.18)	44/197 (22.33)	21/96 (21.88)	
Micropulse laser	23/293 (7.85)	15/197 (7.61)	8/96 (8.33)	
Anti-VEGF injection, n (%)	–	–	29/96 (30.21)	–
Treatment duration (months), median (IQR)	–	–	8 (6–12)	–
Number of injections, median (IQR)	–	–	3 (1.5–5)	–

Symptom duration was defined as the time from the onset of ocular symptoms to inclusion in the study

*MNV* = macular neovascularization; *M* = male; *F* = female; *SD* = standard deviation; *OD* = right eye; *OS* = left eye; *LogMAR* = logarithm of minimal angle of resolution; *BCVA* = best-corrected visual acuity; *IOP* = intraocular pressure; *VEGF* = vascular endothelial growth factor; *IQR* = interquartile range

^*^Statistically significant *P* values are indicated in boldface

^†^Independent sample t-test

^‡^Chi-square test

^§^Fisher’s exact test

Of the 293 enrolled eyes, 197 (67.24%) did not develop MNV and 96 (32.76%) were complicated with MNV. Patients with MNV were significantly older than those without MNV (52.22 ± 8.82 years vs. 48.26 ± 9.06 years, *P* < 0.001). The duration of symptom in the non-MNV group (31.35 ± 31.25 months) tended to be shorter than that in the MNV group (35.66 ± 29.97 months); however, the difference was not statistically significant (*P* = 0.263). Meanwhile, the MNV group exhibited significantly worse BCVA of 0.63 ± 0.47 logMAR compared to 0.33 ± 0.37 logMAR in the non-MNV group (*P* < 0.001). Lateral involvement, spherical equivalent, IOP, and previous treatment were not significantly different between the two groups (all *P* > 0.05). Among the 96 eyes with coexisting MNV, 29 (30.21%) had previously undergone anti-VEGF therapy. The median treatment duration was 8 months (IQR, 6–12 months), and the median number of injections was 3 (IQR, 1.5 to 5).

### Differences in choroidal characteristics between non-MNV and MNV groups

Table 2 shows a comparison of choroidal characteristics between the non-MNV and MNV groups in eyes with chronic CSC. There was no significant difference in the number of VV ampullae in each quadrant or in the total number of ampulla (superotemporal, *P* = 0.648; superonasal, *P* = 0.119; inferotemporal, *P* = 0.291; inferonasal, *P* = 0.242; total, *P* = 0.773). Based on the absence or presence of DVV, choroidal vein drainage pattern can be divided into symmetry and asymmetry. Asymmetric patterns were detected more frequently using WF-ICGA in the MNV group (83/96, 86.46%) than in the non-MNV group (149/197, 75.63%; *P* = 0.032). In addition, there were significant differences in the number and location of DVV between the two groups (*P* = 0.041 and *P* = 0.005, respectively). The presence of one DVV was more frequent in eyes with MNV (67.47%) than in the non-MNV group eyes (53.69%). In eyes with one DVV, the presence of a superotemporal DVV was more frequent in the MNV group than in the non-MNV group (*P* < 0.001). For eyes with two DVVs, there was no difference between the two groups (*P* = 0.946). No significant difference was observed between the two groups in the presence of PVV (*P* = 0.568).

**Table 2:** Comparison of choroidal vasculature characteristics between non-MNV and MNV groups in chronic central serous chorioretinopathy

Choroidal vasculature characteristics	Total	Non-MNV group	MNV group	*P* value^*^
The number of VV ampulla, median (IQR)				
ST	2 (1, 2)	2 (1, 2)	2 (1, 2)	0.648^†^
SN	2 (2, 3)	2 (2, 3)	2 (2, 3)	0.119^†^
IT	2 (2, 3)	2 (2, 3)	2 (2, 3)	0.291^†^
IN	2 (2, 2)	2 (2, 2)	2 (1, 2)	0.242^†^
Total	8 (7, 10)	8 (7, 10)	8.5 (7, 10)	0.773^†^
Choroidal vein drainage pattern, n (%)				**0.032**^**‡**^
Symmetric pattern	61/293 (20.82)	48/197 (24.37)	13/96 (13.54)	
Asymmetric pattern	232/293 (79.18)	149/197 (75.63)	83/96 (86.46)	
Number of DVV, n (%)				**0.041**^**‡**^
One DVV	136/232 (58.62)	80/149 (53.69)	56/83 (67.47)	
Two DVVs	96/232 (41.38)	69/149 (46.31)	27/83 (32.53)	
Location of DVV, n (%)				**0.005**^**§#**^
One DVV				** < 0.001**^**‡**^
ST	41/136 (30.15)	13/80 (16.25)	28/56 (50.00)	
SN	39/136 (28.68)	26/80 (32.50)	13/56 (23.21)	
IT	41/136 (30.15)	30/80 (37.50)	11/56 (19.64)	
IN	15/136 (11.03)	11/80 (13.75)	4/56 (7.14)	
Two DVVs				0.946^§^
ST + SN	28/96 (29.17)	20/69 (28.98)	8/27 (29.63)	
ST + IN	11/96 (11.46)	7/69 (10.14)	4/27 (14.81)	
IT + SN	38/96 (39.58)	28/69 (40.58)	10/27 (37.04)	
IT + IN	13/96 (13.54)	10/69 (14.49)	3/27 (11.11)	
ST + IT	6/96 (6.25)	4/69 (5.80)	2/27 (7.41)	
Presence of PVV, n (%)	18/293 (6.14)	11/197 (5.58)	7/96 (7.29)	0.568^‡^
Presence of IVA, n (%)				
Macular IVA	150/293 (51.19)	92/197 (46.70)	58/96 (60.42)	**0.027**^**‡**^
Peripapillary IVA	72/293 (24.57)	48/197 (24.36)	24/96 (25.00)	0.906^‡^
Temporal IVA	118/293 (40.27)	80/197 (40.61)	38/96 (39.58)	0.867^‡^
Superior IVA	46/293 (15.70)	34/197 (17.26)	12/96 (12.50)	0.293^‡^
Nasal IVA	100/293 (34.13)	69/197 (35.02)	31/96 (32.29)	0.643^‡^
Inferior IVA	22/293 (7.51)	14/197 (7.11)	8/96 (8.33)	0.708^‡^
Presence of ASHS-LIA	27/293 (9.22)	10/197 (5.08)	17/96 (17.71)	** < 0.001**^**‡**^
Grade 1	22/27 (81.48)	8/10 (80)	14/17 (82.35)	0.105^§^
Grade 2	3/27 (11.11)	0/10 (0)	3/17 (17.65)	
Grade 3	2/27 (7.41)	2/10 (20)	0/17 (0)	

*MNV* = macular neovascularization; *VV* = vortex vein; *IQR* = interquartile range; *ST* = superotemporal; *SN* = superonasal; *IT* = inferotemporal; *IN* = inferonasal; *DVV* = dominant vortex vein; *PVV* = posterior vortex vein; *IVA* = intervortex venous anastomosis; *ASHS-LIA* = age-related scattered hypofluorescent spots on late-phase indocyanine green angiography

^#^Comparison between the two groups according to DVV location without stratification by DVV number

^*^Statistically significant *P* values are indicated in boldface

^†^Mann–Whitney U test

^‡^Chi-square test

^§^Fisher’s exact test

In this study, anatomical localization categorized IVAs as macular, peripapillary, or peripheral, depending on early-phase WF-ICGA images. The presence or absence of IVAs was compared between the non-MNV and MNV groups. Compared to that in the non-MNV group, the MNV group exhibited more macular IVA (46.70% vs. 60.42%, *P* = 0.027), whereas no significant differences were observed in terms of the remaining IVA subtypes (peripapillary, *P* = 0.906; temporal, *P* = 0.867; superior, *P* = 0.293; nasal, *P* = 0.643; inferior, *P* = 0.708).

Moreover, late-phase ICGA imaging was used to identify and grade ASHS-LIA in the two groups. ASHS-LIA was identified more frequently in the MNV group (17.71%) than in the non-MNV group (5.08%, *P* < 0.001). However, no significant difference was observed in terms of the grade of ASHS-LIA (*P* = 0.105).

### Choroidal characteristics related to the occurrence of MNV

A generalized linear-mixed model was used to identify whether choroidal vasculature factors were associated with the occurrence of MNV. The results are shown in Table 3. Multivariate analysis identified four independent associated factors for MNV development: presence of superotemporal-DVV (odds ratio [OR]: 14.189; 95% confidence interval [CI]: 5.041–39.943; *P* < 0.001), superotemporal + inferonasal-DVV (OR: 5.179; 95% CI: 1.163–23.058; *P* = 0.031), macular IVA (OR: 2.096; 95% CI: 1.154–3.804; *P* = 0.015), and ASHS-LIA (OR: 24.216; 95% CI: 2.292–110.761; *P* = 0.011). To enhance our model, we incorporated adjustments for age, sex, and symptom duration, which confirmed the significant influence of three characteristics: the presence of superotemporal DVV (*P* < 0.001), macular IVA (*P* = 0.003), and ASHS-LIA (*P* = 0.011). It is worth noting that the high OR for ASHS-LIA was accompanied by a wide CI, likely attributable to the relatively low prevalence of this biomarker in our cohort and should therefore be interpreted with caution.

**Table 3:** Generalized linear-mixed model of the impact of choroidal characteristics associated with macular neovascularization in chronic central serous chorioretinopathy

Choroidal characteristics	Multivariate			Multivariate^a^		
	OR	95% CI	*P* value^*^	OR	95% CI	*P* value^*^
VV ampulla-ST	1.279	0.820–1.995	0.277	1.341	0.824–2.181	0.237
VV ampulla-SN	1.393	0.955–2.034	0.085	1.359	0.903–2.044	0.141
VV ampulla-IT	0.679	0.420–1.097	0.114	0.632	0.383–1.045	0.074
VV ampulla-IN	0.715	0.456–1.123	0.145	0.781	0.475–1.284	0.329
Presence of ST-DVV^b^	14.189	5.041–39.943	** < 0.001**	11.795	3.780–36.810	** < 0.001**
Presence of SN-DVV^b^	1.521	0.566–4.083	0.404	1.548	0.522–4.592	0.429
Presence of IT-DVV^b^	1.914	0.647–5.662	0.239	1.936	0.626–5.987	0.250
Presence of IN-DVV^b^	0.869	0.272–2.777	0.812	0.822	0.259–2.605	0.737
Presence of ST + SN-DVV^b^	2.075	0.630–6.838	0.229	1.987	0.582–6.781	0.272
Presence of ST + IT-DVV ^b^	3.039	0.452–20.432	0.250	3.464	0.474–25.320	0.218
Presence of ST + IN-DVV^b^	5.179	1.163–23.058	**0.031**	2.942	0.480–18.038	0.242
Presence of SN + IT-DVV^b^	1.917	0.594–6.184	0.275	1.558	0.449–5.406	0.483
Presence of IT + IN-DVV^b^	2.685	0.557–12.940	0.217	2.085	0.404–10.765	0.379
Presence of PVV^c^	2.073	0.586–7.331	0.257	1.399	0.412–4.756	0.589
Presence of macular IVA^c^	2.096	1.154–3.804	**0.015**	2.699	1.413–5.155	**0.003**
Presence of peripapillary IVA^c^	1.225	0.572–2.625	0.600	1.565	0.708–3.458	0.267
Presence of temporal IVA^c^	0.909	0.497–1.663	0.755	0.939	0.492–1.791	0.847
Presence of superior IVA^c^	0.590	0.251–1.386	0.225	0.584	0.233–1.463	0.250
Presence of nasal IVA^c^	1.228	0.636–2.372	0.539	1.374	0.687–2.747	0.368
Presence of inferior IVA^c^	1.493	0.447–4.992	0.514	2.110	0.609–7.313	0.238
Presence of ASHS-LIA^c^	24.216	2.292–110.761	**0.011**	33.879	2.418–196.419	**0.011**
Grade of ASHS-LIA	0.328	0.050–2.161	0.245	0.192	0.021–1.720	0.140

*OR* = odds ratio; *CI* = confidence interval; *VV* = vortex vein; *ST* = superotemporal; *SN* = superonasal; *IT* = inferotemporal; *IN* = inferonasal; *DVV* = dominant vortex vein; *PVV* = posterior vortex vein; *IVA* = intervortex venous anastomosis; *ASHS-LIA* = age-related scattered hypofluorescent spots on late-phase indocyanine green angiography

^*^Statistically significant *P* values are indicated in boldface

^a^Enhanced model further adjusted for age, sex, and duration of symptoms

^b^These covariates are binary variables. The absence of a corresponding DVV group was regarded as reference

^c^These covariates are binary variables. Groups without PVV, IVA, or ASHS-LIA were regarded as reference groups

### Interobserver agreement

Interobserver agreement for WF-ICGA-derived choroidal characteristics was quantified using ICC and kappa coefficient. The ICCs for the number of VV ampulla in the superotemporal, superonasal, inferotemporal, and inferonasal quadrants were 0.91, 0.87, 0.93, and 0.95, respectively. The kappa coefficients for the location of DVV, presence of PVV, and ASHS-LIA were 0.85, 0.95, and 0.92, respectively. The kappa coefficients for the presence of IVAs located in the macular region, around the optic disc, temporal, superior, nasal, and inferior were 0.83, 0.88, 0.84, 0.90, 0.91 and 0.95, respectively. These data reflected a high degree of concordance between the investigators, and interobserver discrepancies were resolved through adjudication.

## Discussion

In this retrospective observational study, we evaluated whether choroidal vasculature biomarkers were associated with MNV formation in eyes with chronic CSC. Our study showed a 32.76% prevalence of MNV among eyes with chronic CSC, with the MNV group presenting with older age and worse visual function. In affected eyes with secondary MNV, the choroidal vasculature was more frequently characterized by an asymmetric distribution pattern, a higher prevalence of solitary DVV, superotemporal quadrant-located DVV, and an increased incidence of macular IVA and ASHS-LIA. The presence of the superotemporal DVV, macular IVA, and ASHS-LIA may serve as independent factors associated with MNV development.

Although chronic CSC generally has a favorable prognosis, it could progress to a sight-threatening condition and result in legal blindness [25]. The occurrence of MNV served as one of the most common sequela of CSC, with a reported prevalence ranging from 15.6% to 34.5% [9, 20, 25, 26], consistent with our findings. Reportedly, the presence of MNV was significantly correlated with worse visual acuity at the final follow-up [4, 25]. Unlike the conservative approach typically employed for CSC, the detection of complicated MNV necessitated prompt initiation of anti-VEGF therapy, reflecting a shift in the therapeutic regimen [5, 27]. Consequently, identifying the risk factors and elucidating the mechanisms responsible for MNV formation represent a crucial objective.

The pathogenesis of CSC remains incompletely understood, although prior research has suggested a potential link to VV congestion, specifically referring to choroidal venous overload [10, 28]. The concept of venous overload has been elucidated through collective evidence. Spaide et al. [10, 23] documented the phenomenon of IVA among pachychoroid spectrum, which further supported the existence of compensatory mechanisms and collateral circulation. Hiroe et al. reported that congestion in DVV area led to increased vascular permeability within the fenestrated choriocapillaris of the macular region [29]. This theoretical framework was also corroborated by additional imaging characteristics, such as choriocapillaris filling delay and dilated larger choroidal vessels [12], and these clinical findings had been consistently replicated in monkey models [30]. The pathogenesis of MNV formation in chronic CSC was speculated to involve a cascade of pathological events, notably outer retinal hypoxia, subsequent RPE dysfunction, and the breakdown of Bruch’s membrane, which was similar to those in AMD [5, 9, 25]. Additionally, Cennamo et al. [16] investigated the choriocapillary vascular density in CSC-associated MNV eyes and proposed that a choriocapillary hypoperfusion may be related to the subsequent progression to MNV. In agreement with the findings reported by Viggiano et al. [31], the choriocapillaris-Sattler complex was significantly thinner in CSC eyes complicated by type 1 MNV than in uncomplicated CSC eyes. Our team’s prior research demonstrated a significant reduction in CCFD in the asymmetric group with a single DVV compared to that of those with symmetric pattern and asymmetric group with two DVVs [13]. We therefore hypothesize that specific choroidal vascular phenotypes predispose eyes to MNV development.

Several scientific investigations have identified various key risk factors for the development of MNV in patients with CSC. Specifically, advanced age and chronic course of disease have been acknowledged as predictors for MNV development in terms of demographic variables [4, 9]. Several OCT biomarkers have been recognized as risk factors for the co-existence of MNV, including intraretinal hyperreflective foci, inner choroidal attenuation, double-layer sign, and inner choroidal fibrosis, etc. [8, 32, 33]. With respect to genetic predisposition, *ARMS2*, *CFH*, *COL4A3*, and *B3GALTL* have been recognized as susceptibility genes for MNV [34]. Nevertheless, limited research has focused on how WF-ICGA-derived choroidal vascular characteristics relate to MNV development. Our study aims to address this knowledge gap.

The present study reported that asymmetric distribution was significantly more pronounced in eyes in the MNV group than in the eyes in the non-MNV group (*P* = 0.032). Mori et al. demonstrated that asymmetric drainage observed in approximately 50% of normal eyes, maintained a consistent prevalence across age groups, suggesting this anatomical variant was neither pathological nor age-dependent [35]. A higher prevalence of asymmetric drainage was observed among CSC cases [13]. But there was no discernible difference in choroidal venous drainage routes between acute and chronic presentations (MNV-absent), further indicating this characteristic not attributable to the duration of symptom [36]. Our finding could be explained by the inference that an imbalance in choroidal venous drainage led to compromised outflow and resultant choroidal venous insufficiency [18]. Of these, the asymmetric phenotype with single DVV was prone to be accompanied by MNV compared to those with two DVVs (*P* = 0.041). A previous investigation identified reduced CCFD in eyes with a single DVV [13], and this hypoperfusion was implicated in the subsequent progression to MNV [16], further verifying our findings.

Furthermore, the superotemporal DVV pattern was more prevalent in the eyes with chronic CSC-associated MNV (*P* = 0.005). Multivariate analysis confirmed its presence as an independent factor associated with MNV development (*P* < 0.001). This observation may be interpreted as venous overload and inter-quadrant heterogeneity. First, the macular region with maximal blood flow is primarily drained by temporal VVs, with high perfusion susceptible to hemodynamic stasis [18]. Second, compared to the inferior drainage pathway, whose outflow is facilitated by gravity, the superior pathway works against gravity, resulting in a higher outflow resistance and a predisposition to obstruction. This can cause excessive blood accumulation within the Haller’s layer, which in turn compresses the superficial choriocapillaris and induces capillary ischemia. The results of Funatsu et al.’s [37] study confirmed this hypothesis that macular choroidal thickening was more pronounced in CSC eyes with a superior-dominant pattern. Third, the existing literature revealed that compared to healthy eyes, the superotemporal VV in pachychoroid eyes occupied a farther and higher position, located farthest from the optic disc with the largest angular displacement from the fovea-disc line [38]. The extended drainage pathway imposed greater demand on choroidal perfusion to overcome gravitational resistance, making it prone to stasis [38].

Macular IVA is another choroidal feature associated with the occurrence of MNV. The pathological significance of IVA, which was recognized as a compensatory collateral vessel in CSC, has not been fully elucidated, although previous studies have documented various location-specific IVAs [23]. Spaide et al. [23] observed that anastomotic connections were more prevalent in the macular region among CSC and pachychoroid-associated neovascularization cases, whereas a peripapillary distribution was dominant in peripapillary pachychoroid syndrome. Our CSC cohort showed that the prevalence of macular IVA was approximately twice that of peripapillary IVA, further confirming the aforementioned observation. The formation of collateral vessels serves as a compensatory mechanism in venous overload theory. Our analysis revealed no significant differences in the remaining IVAs in other locations between the two cohorts (all *P* > 0.05), with the exception of macular IVA. Compared to MNV-absent eyes, eyes with chronic CSC-associated MNV had more macular IVA (*P* = 0.027), which was another factor correlating with the formation of MNV (*P* = 0.003). Our study highlights the differential pathological significance of IVAs based on their anatomical locations, with macular IVA warranting closer monitoring. Miyara et al. [15] reported that the non-watershed group, in which the horizontal watershed was ill-defined due to the existence of marked anastomoses, had more CVH areas than that of the watershed group, with VV anastomosis indicating severe choroidal congestion. Consequently, we hypothesized that the presence of macular IVA indicates intense engorgement, thereby exerting mechanical compression on the choriocapillaris and inducing choroidal ischemia. This condition triggers the formation of pathological neovascularization to nourish the outer retina and RPE [8]. Owing to the inherent limitations of this cross-sectional study, the possibility that macular IVA represents a secondary alteration cannot be excluded. The high metabolic demand of the macular region renders it particularly susceptible to ischemia, and the formation of compensatory anastomoses may represent an adaptive response to improve perfusion [18]. Consequently, the temporal relationship between macular IVA and MNV warrants further investigation in longitudinal studies.

Interestingly, the presence of ASHS-LIA was more frequent in the MNV group than in the non-MNV group (*P* < 0.001), which was the third associated factor for the development of neovascularization (*P* = 0.011). The existing literature indicates that ASHS-LIA demonstrates bilateral presentation and correlates with demographic variables, including age and sex [24, 39]. After adjusting for these confounders, ASHS-LIA remained a significant biomarker for MNV. However, the underlying mechanism requires further elucidation. During late-phase ICGA, RPE cells are capable of taking up ICG molecules and contributing to fluorescence signals [40, 41], whereas ASHS-LIA may reflect RPE dysfunction, which is involved in the development of MNV. Emerging evidence has indicated that ASHS-LIA may be involved in the pathogenesis of PCV [42, 43]. Moreover, given that both PCV and CSC belong to pachychoroid diseases, it is plausible that they share overlapping pathogenic mechanisms [14]. Currently, ASHS-LIA was usually recognized as a precursor of basal linear deposit (BlinD) [24]. Curcio et al. demonstrated that BlinD consisted of a thin, compact layer of lipoprotein-derived debris located between the RPE basal lamina and the inner collagenous layer of Bruch’s membrane, which might impede oxygen diffusion from the choroid to the RPE cells [24, 42, 44]. Consequently, in the already hypoxic microenvironment of chronic CSC, this lipoprotein deposition could exacerbate hypoxia in RPE cells, potentially inducing VEGF secretion and thereby promoting neovascularization [42]. Furthermore, Kim et al. [45] identified ASHS-LIA as a marker of adverse prognosis in PCV, related to compromised disease stability, elevated recurrence rates, and greater therapeutic demands. Combined with our findings, the clinical utility of ASHS-LIA warrants continued attention in patient management.

This study had some limitations. First, given that the macular IVA may represent a compensatory response to MNV, the retrospective cross-sectional nature of our study could not establish a definitive causal relationship, requiring validation through prospective longitudinal investigations. Second, the pathophysiological explanations proposed for the observed angiographic features, which are based on the current understanding of pachychoroid disease, remain hypothetical and require validation through histopathological studies. Third, the degree of subjectivity cannot be entirely excluded from the evaluation of primary choroidal features. Fourth, although the study enrolled a substantial cohort, the relatively low prevalence of certain features, such as PVV and ASHS-LIA, necessitated further validation through expanded sample sizes.

## Conclusion

Despite these limitations, our study revealed several choroidal imaging biomarkers associated with MNV formation in chronic CSC. Asymmetric distribution, solitary DVV, superotemporal quadrant-located DVV, macular IVA, and ASHS-LIA were more prevalent in patients with chronic CSC-associated MNV than in those without. More importantly, the presence of superotemporal DVV, macular IVA, and ASHS-LIA was identified as independent associated factors for the occurrence of MNV in eyes with chronic CSC. Our study provides a novel investigation concentrating on the association of WF-ICGA-derived choroidal vascular characteristics with MNV, further shedding new light on MNV formation in chronic CSC and supporting enhanced surveillance for high-risk patients.

## Data Availability

No datasets were generated or analysed during the current study.

## Abbreviations

AMD: Age-related macular degeneration
ASHS-LIA: Age-related scattered hypofluorescent spots on late-phase indocyanine green angiography
BCVA: Best-corrected visual acuity
BlinD: Basal linear deposit
CCFD: Choriocapillaris flow density
CI: Confidence interval
CSC: Central serous chorioretinopathy
CVH: Choroidal vascular hyperpermeability
DVV: Dominant VV
FFA: Fundus fluorescein angiography
FIPED: Flat, irregular pigment epithelium detachment
ICC: Intraclass correlation coefficient
ICGA: Indocyanine green angiography
IOP: Intraocular pressure
IQR: Interquartile range
IVA: Intervortex venous anastomosis
MNV: Macular neovascularization
OCT: Optical coherence tomography
OR: Odds ratio
PCV: Polypoidal choroidal vasculopathy
PVV: Posterior VV
RPE: Retinal pigment epithelium
SD: Standard deviation
SRF: Subretinal fluid
UWF-ICGA: Ultra-widefield indocyanine green angiography
VEGF: Vascular endothelial growth factor
VV: Vortex vein
WF-ICGA: Widefield indocyanine green angiography
